# Semi‐supervised determination of pseudocryptic morphotypes using observer‐free characterizations of anatomical alignment and shape

**DOI:** 10.1002/ece3.3058

**Published:** 2017-06-02

**Authors:** Natasha S. Vitek, Carly L. Manz, Tingran Gao, Jonathan I. Bloch, Suzanne G. Strait, Doug M. Boyer

**Affiliations:** ^1^ Florida Museum of Natural History University of Florida Gainesville FL USA; ^2^ Department of Biology University of Florida Gainesville FL USA; ^3^ Department of Genetics, Development, and Cell Biology Iowa State University Ames IA USA; ^4^ Department of Mathematics Duke University Durham NC USA; ^5^ Department of Biological Sciences Marshall University Huntington WV USA; ^6^ Department of Evolutionary Anthropology Duke University Durham NC USA

**Keywords:** Erinaceomorpha, fossil, high‐throughput, Marsupialia, molar, morphology, morphometrics, *Mus musculus*, phenomics, phenotype, simulation

## Abstract

Accurate, quantitative characterization of complex shapes is recognized as a key methodological challenge in biology. Recent development of automated three‐dimensional geometric morphometric protocols (auto3dgm) provides a promising set of tools to help address this challenge. While auto3dgm has been shown to be useful in characterizing variation across clades of morphologically very distinct mammals, it has not been adequately tested in more problematic cases where pseudolandmark placement error potentially confounds interpretation of true shape variation. Here, we tested the sensitivity of auto3dgm to the degree of variation and various parameterization settings using a simulation and three microCT datasets that characterize mammal tooth crown morphology as biological examples. The microCT datasets vary in degree of apparent morphological differentiation, with two that include grossly similar morphospecies and one that includes two laboratory strains of a single species. Resulting alignments are highly sensitive to the number of pseudolandmarks used to quantify shapes. The degree to which the surfaces were downsampled and the apparent degree of morphological differentiation across the dataset also influenced alignment repeatability. We show that previous critiques of auto3dgm were based on poorly parameterized alignments and suggest that sample‐specific sensitivity analyses should be added to any research protocol including auto3dgm. Auto3dgm is a useful tool for studying samples when pseudolandmark placement error is small relative to the true differences between specimens. This method therefore represents a promising avenue forward in morphometric studies at a wide range of scales, from samples that differ by a single genetic locus to samples that represent multiple phylogenetically diverse clades.

## 1 INTRODUCTION

A major challenge for evolutionary biologists, including paleontologists and paleoanthropologists, is to understand how morphological diversity can inform our understanding of micro‐ and macroevolution. Hypotheses of tempo and mode of evolution, patterns and drivers of diversification, and adaptation all depend on an accurate characterization of anatomical form (Eldredge & Gould, 1972; Gingerich, 1974a; Hunt, 2007; Losos, 1998; Mullen & Hoekstra, 2008; Pinkham 1971). Additionally, discriminating shape is an integral component of delimiting species based on anatomical features (De Queiroz, 2007; Sáez et al., 2003).

In this context, quantitative description of biological shape ideally should (i) be carried out using homologous anatomical elements, (ii) sufficiently represent the majority of difference between objects, and (iii) be repeatable. For example, characterizations of tooth crown morphology are useful for delimiting mammalian species by size in the fossil record in part because teeth show little size variation due to ontogeny and have well‐characterized ranges of size variation within species (Gingerich, 2014). Simple linear measurements of teeth have been widely and effectively used by paleobiologists to provide quick approximations of overall tooth size and tooth dimensions (e.g., ratio of length to width) in relatively large samples. In turn, these sizes and ratios have been used to differentiate between species that otherwise have similar tooth morphologies (Barnosky, 1990; Benazzi et al., 2011; Gingerich, 1974a, 1974b; Guthrie, 1965; Pinkham 1971). However, quantifications of shapes that might more closely approach the true complexity and variability of tooth crown surfaces are more elusive. When interspecific linear dimensions are similar and intraspecific variation in shape is high relative to interspecific variation, broad patterns in tooth morphology and their correspondence to species boundaries can be difficult to assess with simple linear measurements alone (Carrasco, 1998). Furthermore, quantitatively characterizing broad, macroevolutionary patterns in dental morphology can be further stymied when measurements are limited by ambiguity or lack of character homology between disparate forms (Polly, 2008a).

Other studies of variation in teeth have used a range of methods, including (i) qualitative or semi‐quantitative methods to assess discrete features (Barnosky & Bell, 2003; Oppenheimer, 1965), (ii) quantitative methods to assess two‐dimensional outline or occlusal features based on Fourier descriptors (Renaud, 2005), eigenshape analysis (Polly, 2003), or geometric morphometrics (McGuire, 2010; Wood et al., 2007), or (iii) quantitative methods to assess univariate 3D topographic measures of a single surface on a tooth or bone (Boyer, 2008; Boyer, Evans, & Jernvall, 2010; Chester, Bloch, Secord, & Boyer, 2010; Evans, Wilson, Fortelius, & Jernvall, 2007; Pampush et al., 2016; Polly, 2008b). However, these methods are more time‐intensive to use than simple linear measurements. Many methods can only be applied to homologous features that are present on all specimens, excluding features that may be important sources of intraspecific variation, such as cuspules on molars, and limiting their potential to quantitatively represent broadly disparate shapes (Polly, 2008a).

Recent interdisciplinary work between comparative anatomists and mathematicians has produced a new class of morphometric protocols (Boyer et al., 2011, 2015a; Boyer, Winchester, & Kay, 2015b; Gao et al. in review) that are designed to reduce the time, subjectivity, and idiosyncrasies of user‐determined measurements, as well as to provide quantifications that simultaneously include information about shape diversity and shape disparity. Specifically, these new methods include automatic alignment of all study objects and computation of Procrustes distances between them based on a predetermined number of evenly spread pseudolandmarks (Boyer et al., 2015a, 2015b). The geometric algorithms utilize operational heuristic cognitive processes that anatomists themselves have traditionally used to align and compare homologous structures between morphologically different species (Boyer et al., 2015a). The pseudolandmark datasets and Procrustes distance matrices that result can then be analyzed according to procedures already in standard use (Zelditch, Swiderski, Sheets, & Fink, 2012). We refer to these methods as “observer‐free” in regard to automated placement of pseudolandmarks and alignment.

Several studies published to date using this newer class of methods successfully recovered the same morphologic groups recognized by user‐determined morphometrics (Boyer et al., 2011, 2015a; Gao et al. in review). However, one in which intergroup differences were small compared to intragroup variation was less successful (Gonzalez, Barbeito‐Andrés, D'Addona, Bernal, & Perez, 2016), suggesting that the performance of these methods in samples such as this requires further testing. In particular, the amount of pseudolandmark placement error (PPE) inherent to the auto3dgm algorithm may impede the method's ability to accurately characterize shape. Samples with relatively little phenotypic variation correlated with a variable of interest, either because that variation is in shape subregions that are physically small compared to the entire area or because the signal of interest is potentially outweighed by intragroup variability, have remained stubborn problems in the study of evolution (Sáez et al., 2003). Despite the initially disappointing results of Gonzalez et al. (2016), we feel that these new morphometric protocols are a promising tool for studying such difficult morphological systems because of both their ability to capture a high degree of morphological detail and their high‐throughput approach.

**Figure 1:**
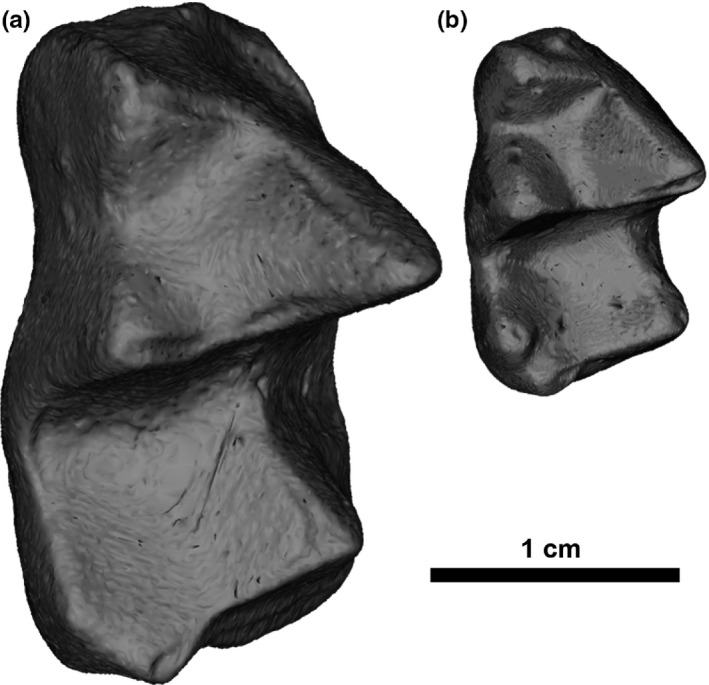
Occlusal view of second or third lower molars (M/2 or M/3) of the two taxa included in the marsupial dataset; (a) *Mimoperadectes labrus*, UF 251318; (b) *Peradectes protinnominatus*, UF 326912

**Figure 2:**
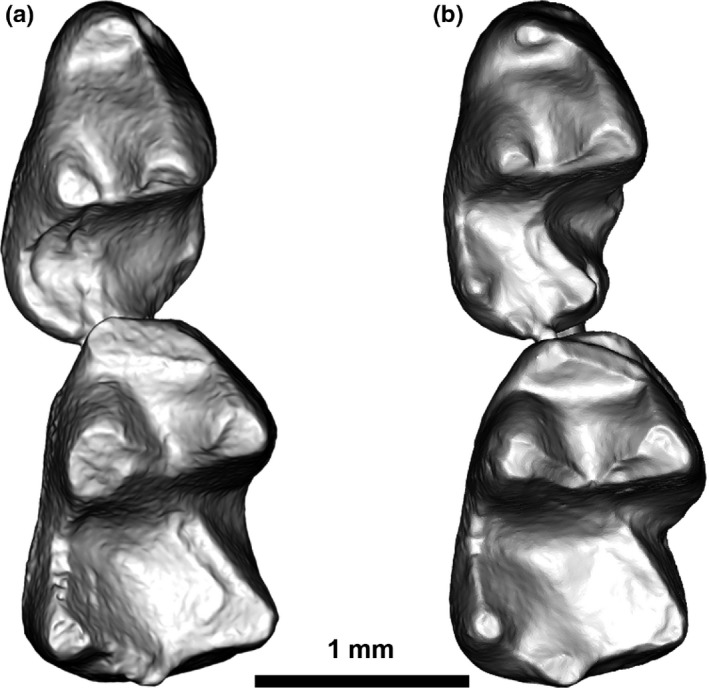
Occlusal view of partial lower jaws containing fourth lower premolars and first molars (P/4‐M/1) of the two taxa included in the erinaceomorph dataset; (a) *Macrocranion junnei*, UCMP 223729; (b) cf. *Colpocherus* sp., UF 253591

Here, we further test the sensitivity of auto3dgm and its suitability for studying the structure of morphological variation in cases where PPE may obscure true patterns of variation. We first discuss PPE and analyze a simulated dataset which demonstrates the effect of PPE when characterizing identical shapes in comparison with increasingly different shapes. We then apply similar analyses to three biological datasets. Those datasets range in phylogenetic and temporal scale from pairs of closely related, extinct species (Figures 1 and 2) to previously studied samples of laboratory strains of *Mus musculus* used by Gonzalez et al. (2016).

Institutional abbreviations are as follows: UCMP—University of California Museum of Paleontology, Berkeley, CA; UF—Florida Museum of Natural History, Vertebrate Paleontology, Gainesville, FL.

## 2 MATERIALS AND METHODS

### 2.1 Description of alignment algorithm

The goal of auto3dgm is to place geometric morphometric pseudolandmarks on 3D objects without human input. Therefore, the algorithm both needs to identify corresponding pseudolandmarks across a set of specimens and also minimize the Procrustes distance between the specimens in a large dataset (10 < *N* < 500). Making such an algorithm converge is a challenge because there are many reasonable solutions for a given dataset. Additional unreasonable solutions may emerge from the optimization procedure. The results of repeated alignments of the same dataset can be compared to evaluate the range of solutions produced by auto3dgm with a given combination of input and settings.

In each alignment, the algorithm starts by randomly placing two sets of pseudolandmarks on the surface of each specimen, one called the initial set and the other the final set. The numbers of pseudolandmarks in each set are specified by the user, and the size of the initial set is often smaller than the final set. In the first stage, the algorithm aligns each pair of surfaces using only the initial pseudolandmarks. It uses the output optimal alignment to compute a Generalized Dataset Procrustes Distance (Puente, 2013) between two sets of pseudolandmarks. After finishing all pairwise alignments, a Minimum Spanning Tree (MST) for the whole collection is constructed, which connects pairs of specimens via paths in the tree. Those paths are used to improve the initial alignment by allowing the algorithm to quickly traverse the dataset with the goal of minimizing the total edge distance on the tree. That transitive alignment acts as a good initial starting point for the second stage of the algorithm. In the second stage, the algorithm essentially repeats the procedures in the first stage using the final set of pseudolandmarks. The transitive alignment using the final, larger set of pseudolandmarks is more accurate than the alignment computed using the initial set of landmarks, and allows for a more accurate alignment of the original input surfaces (Boyer et al., 2011, 2015a, 2015b; Puente, 2013).

### 2.2 Source of pseudolandmark placement error

It is important to note that unlike semilandmarks, the pseudolandmarks used by the auto3dgm algorithm do not slide across the surface once they are placed. Therefore, a certain amount of random pseudolandmark placement error (PPE) is introduced at the very beginning of the algorithm when the two sets of pseudolandmarks are sampled from the original specimen using a farthest point sampling scheme (Boyer et al.2015a). The PPE is one kind of measurement error, similar to human landmark placement error and other kinds of digitization error in geometric morphometric analyses (Fruciano, 2016). If the difference between corresponding pseudolandmarks on different specimens is less than the differences between the underlying surfaces that are sampled by those pseudolandmarks, then this PPE will contribute an acceptable, relatively small amount of random shape variation, or “noise” to the total amount of variation within the pseudolandmark datasets. However, a higher relative contribution of PPE to the total variation in the dataset can lead to unacceptable alignments and meaningless outputs of pseudolandmark clouds. Unacceptably high amounts of PPE are more likely to occur (i) when alignment settings include a low number of final pseudolandmarks, which increases the amount of PPE, and (ii) when the underlying shapes of interest are highly similar to each other, which decreases the true differences sampled by those pseudolandmarks.

### 2.3 Datasets

**Figure 3:**
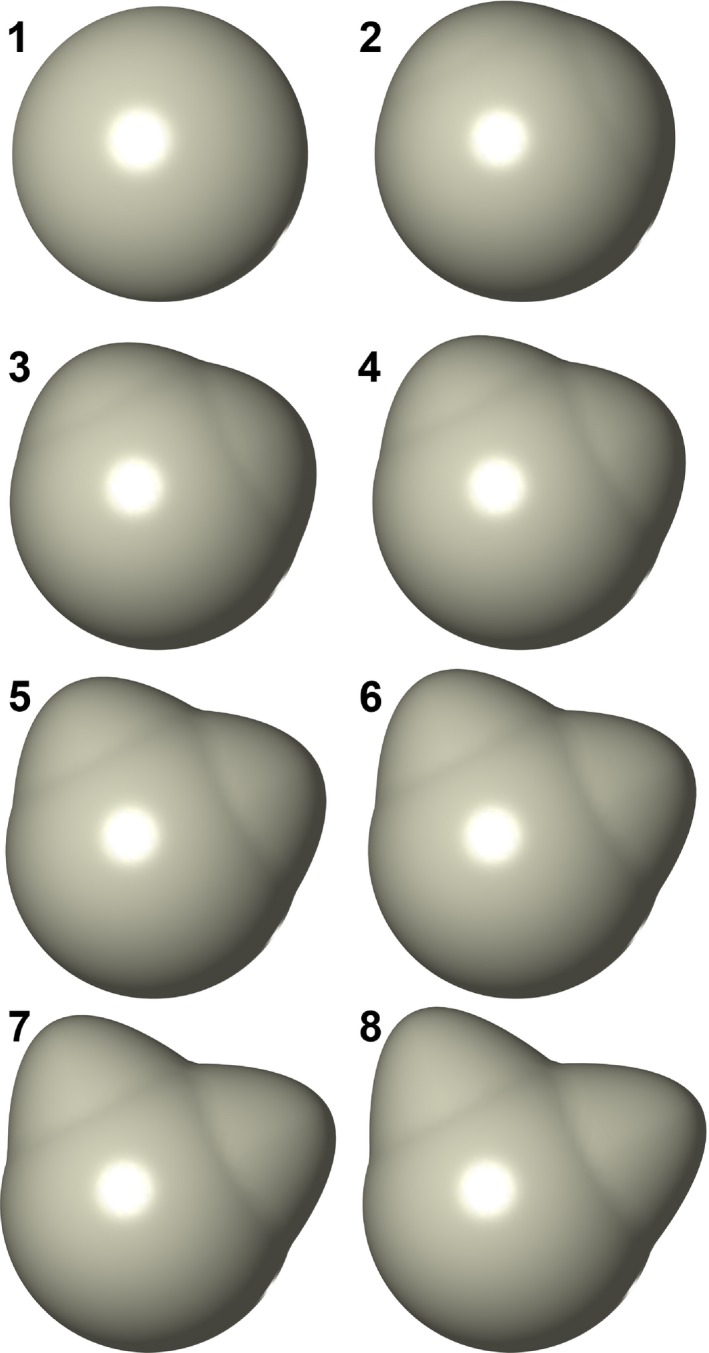
Shapes used in the simulated dataset. Numbers next to each shape refer to their identity in the principal components plots in Figure 8

One simulated dataset and three biological datasets were analyzed with the goal of characterizing the relative contribution of (i) pseudolandmark numbers and (ii) differences between underlying shapes to the acceptability of an aligned dataset. The simulated dataset was designed to test two expectations of well‐chosen alignment settings with acceptably low PPE (Figure 3). First, identical specimens should occupy slightly different positions in Euclidean tangent shape space due to PPE alone, even in acceptable alignments. Second, that variation in the position of identical specimens should not overlap with nonidentical specimens in an acceptable alignment. To assess the first expectation, six identical copies of each of two shapes (shapes 1 and 8, Figure 3) are included in the simulated dataset. One replicated shape is a standard unit sphere {(*x*,*y*,*z*): *x* ^2^ + *y* ^2^ + *z* ^2^ = 1}. The other replicated shape is built upon the same sphere but with two additional symmetrical hemi‐ovoids centered at (0,0,1) and (0,1,0), respectively; each hemi‐ovoid is created by first picking its center and reading off the surface normal direction at the center, then translating points on the sphere within geodesic distance π/4 to the center by an amount inversely proportional to the geodesic distance along the direction of the surface normal at the center.

To assess the second expectation, we include an additional six shapes that fall along a continuum between the two end‐member shapes (total *N* = 18). In total, the dataset has a single, unidirectional pattern of true shape variation and we expect that the first principal component alone can adequately summarize the shape variation of interest. For unacceptable alignments, we expect notable overlap along the first principal component between the morphospaces occupied by the identical shapes and the nonidentical, intermediate shapes. Correspondingly, alignments of better quality should lead to less significant overlaps between the two morphospaces. An ideal alignment should result in a predictable organization of shapes in principal component space: Each set of identical shapes should occupy a distinct cluster at either extreme of the first principal component, and the intermediate shapes should be ordered and relatively evenly spaced between those two clusters.

Specimens for the first two biological datasets were chosen from a collection of fossil mammal teeth from the earliest Eocene of the Bighorn Basin, Wyoming. The collection is part of one of the best sampled terrestrial isotopic and fossil records of the Paleocene‐Eocene Thermal Maximum (Baczynski et al., 2013; Bowen, Koch, Gingerich, Bains, & Corfield, 2001; Gingerich, 1989; Kraus et al., 2013). During that interval 56–55.8 million years ago, global and regional climate shifted by 5–8°C (Bowen et al., 2001; Westerhold, Röhl, McCarren, & Zachos, 2009; Zachos, 2001). This notable climatic aberration was accompanied by geographic range shifts in the flora, permanent reorganization of the fauna, and body size changes in multiple vertebrate lineages (Chester et al., 2010; Clyde & Gingerich, 1998; Secord et al., 2012; Smith, 2009; Wing et al., 2005). Against the backdrop of that environmental and ecological change, assessing potential changes in morphology on the scale addressed by this study is a primary current research interest. The collection was considered representative of the kinds of datasets of fossils likely to be used with auto3dgm but potentially susceptible to overly high PPE.

The first of those two datasets consists of equal numbers of lower molars from each of two marsupial taxa, *Peradectes protinnominatus* and *Mimoperadectes labrus* (*n* = 15 each, total *n* = 30). Because fossil teeth are often recovered as isolated specimens and the morphology of the second and third lower molars (M/2s and M/3s) of these taxa are very similar, these tooth positions could not be differentiated and were therefore pooled for each species. The practice is common for isolated marsupial molars (e.g., Rose et al., 2012; Smith, 2007; Williamson & Taylor, 2011), and the exact tooth position of each specimen was not relevant to determining alignment repeatability. Specimens were identified as *P. protinnominatus* or *M. labrus* and discriminated from a third marsupial present in the assemblage, *Herpetotherium innominatum*, using published descriptions and analyses (Rose et al., 2012; Smith, 2007; Strait, 2001). *Mimoperadectes labrus* and *P. protinnominatus* have similar, conservative tribosphenic lower molar morphology (Gordon, 2003). It is worth noting that the two taxa can be discriminated based on size alone because the lower molars of *M. labrus* are approximately 150% as long as those of *P. protinnominatus* (Figure 1). However, this size discrepancy is not utilized by auto3dgm in the quantification of shape variation because all specimens are scaled to identical centroid size before automated alignment begins. Size provides an independent check on whether the variation between the shape of the two marsupial taxa is one of the primary sources of variation in a given auto3dgm alignment. If it is, then the two taxa should be separated along the primary axis resulting from a principal components analysis.

The second dataset consists of first and second molars (M/1s and M/2s, *n* = 42) of primitive erinaceomorph insectivores identified as either *Macrocranion junnei* or cf. *Colpocherus* sp. These two taxa have been distinguished based on lower fourth premolar (P/4) morphology, but the differences in molar morphology are much more subtle (Rose et al., 2012). In our study, independent observers could not reliably distinguish isolated molars a priori based on previously published descriptions (Rose et al., 2012; Smith, Bloch, Strait, & Gingerich, 2002; Strait, 2001). Two specimens were associated with P/4s, and only those two specimens were positively identified to either taxon before analysis (Figure 2).

A third biological dataset, comprised of microCT scans of molar rows (*n* = 19) of extant laboratory strains of *Mus musculus* that were used by Gonzalez et al. (2016), was also aligned and analyzed. The two groups differ by a single genetic locus. An experimental group was homozygous for a mutation resulting in deficient growth hormone levels. A control group was heterozygous for the same mutation, which resulted in normal growth hormone levels. Scan settings and further details can be found in Gonzalez et al. (2016).

All fossil specimens were microCT‐scanned on Nikon XTH 225 ST at the Shared Materials Instrumentation Facility at Duke University, NC. Specimens were scanned at 3.7–6.6 μm resolution at scan intensity settings KV = 125, μAmp = 33–41. Voxel sizes for specimens were calibrated using ceramic spheres of known diameter scanned three times at the same settings as the specimens. Digital crown surfaces for each fossil specimen were extracted using Avizo 8.1 (Visualization Science Group) and cropped by hand at the cervical margin of the crown.

### 2.4 Pseudolandmark and mesh settings

Datasets were repeatedly aligned using auto3dgm for both R 3.0.2 and MATLAB R2015A (Boyer et al. 2015a, 2015b; Puente, 2013; R Core Team 2015). Nine replicate alignments were generated for each combination of the parameters listed below for the simulation, marsupial, erinaceomorph, and *M. musculus* dataset. The surfaces used for alignment were either at full resolution or were downsampled to 100,000, 50,000, 10,000, or 5,000 triangles per mesh (100k, 50k, 10k, or 5k) using batch processing in MeshLab. The surfaces in the simulated dataset and *Mus musculus* dataset were already at a relatively low resolution (8,192 and 1,500–6,500 faces, respectively). They were not further downsampled. Each alignment was performed with the initial number of pseudolandmarks set to 64, 128, 256, or 256 points and the final number of pseudolandmarks set to 128, 256, 512, or 1,024 points, respectively. The simulation dataset was additionally aligned at the following initial and final pseudolandmark settings: (512/2048), (512/4,096). In order to replicate previously published analyses as closely as possible and then to extend them, we aligned the *M. musculus* specimens at the following initial and final pseudolandmark settings: (200/399), (250/600), (800/1,000), (1,000/2000), (1,500/3,000). A principal components analysis (PCA) was performed on the point clouds of aligned specimens produced by each alignment replicate for all datasets.

Repeatability in output data structure was measured in four ways. First, for all datasets, we calculated the correlation coefficient (*R* ^2^) of first principal component (PC 1) scores from pairs of PCAs for all combinations of replicates at a given input setting (Boyer et al., 2015a). Second, in contrast to the reduced proportion of variation explained by PC1, we performed Mantel tests (permutations = 999) between all pairwise combinations of replicates for a given input setting using pseudolandmark coordinates as data. For each test, we calculated the observed correlation statistic (*r*) and its quantile among the permutations (*p*‐value). The Mantel test function was modified for use with 3D shape coordinates following Claude (2008). Third, hierarchical clustering was performed using the UPGMA method, which had the highest cophenetic correlation coefficient of the available methods (Zelditch et al., 2012). Dendrograms produced by different replicates at a given input setting were compared using Robinson‐Foulds distances (Robinson & Foulds, 1981). Fourth, for the simulated dataset only, we used Procrustes ANOVA calculations of repeatability (Fruciano, 2016; Klingenberg & McIntyre, 1998). Given that some amount of PPE will be present in all replicates, we did not expect that the Mantel tests correlations would be at or near one, nor did we expect the Robinson‐Foulds distances to necessarily converge on zero. However, Mantel test *p*‐values and Robinson‐Foulds distances should generally decrease with increasing repeatability of alignments. Additionally, we expected that in suitable alignments, repeatability measures for PC1 scores should be higher than repeatability of the dataset as a whole based on the expectation that the primary patterns of variation in each dataset should be reproducibly recovered despite the presence of noise in the dataset.

For each of the possible settings per dataset (simulation = 6, fossils = 20, *Mus musculus *= 5), values for each of the four tests were calculated and distributions of those values were examined. Because distributions were not always normal and often bimodal, both mean and median values were calculated for each parameter setting for all datasets, along with standard deviation, quartile, and range. Confidence intervals were based on interquartile range instead of standard deviation because of the range of distributions. Pairwise *t*‐tests were performed on mean values and nonparametric Mann–Whitney *U*‐tests were performed on median values to test for significantly different performance between different parameter settings. Outputs of both tests are reported with Bonferroni's corrections. Summary statistics and tests for performance differences are reported in the Supplementary Data.

The nine replicates were also used to calculate median absolute deviation for each specimen in the first two principal components as a measure of how alignment error was distributed across specimens. Median absolute deviation was used instead of the related, and more common, metric mean standard deviation because the distribution of positions in the first two axes of principal component space was not consistent and often not normal.

**Figure 4:**
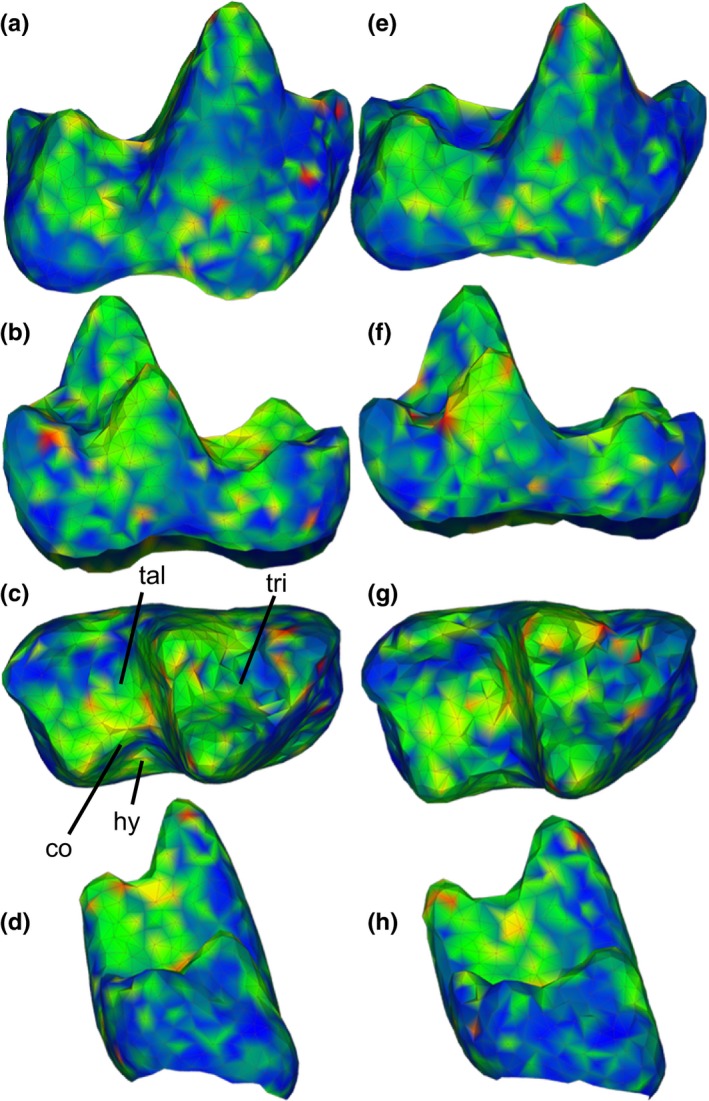
Shapes represented by the extremes of the first principal component (PC 1) of a dataset of two marsupial taxa; (a) buccal, (b) lingual, (c) occlusal, and (d) posterior views of the minimum of PC 1; (e–h) same views of the maximum of PC 1. Shapes also correspond to shape differences between the marsupial species *Mimoperadectes labrus* (minimum) and *Peradectes protinnominatus* (maximum). Shapes are colored by the squared Euclidean distance between each point in the two surfaces. Warmer colors indicate greater distances. co, crista obliqua; hy, hypoflexid; tal, talonid; tri, trigonid

**Figure 5:**
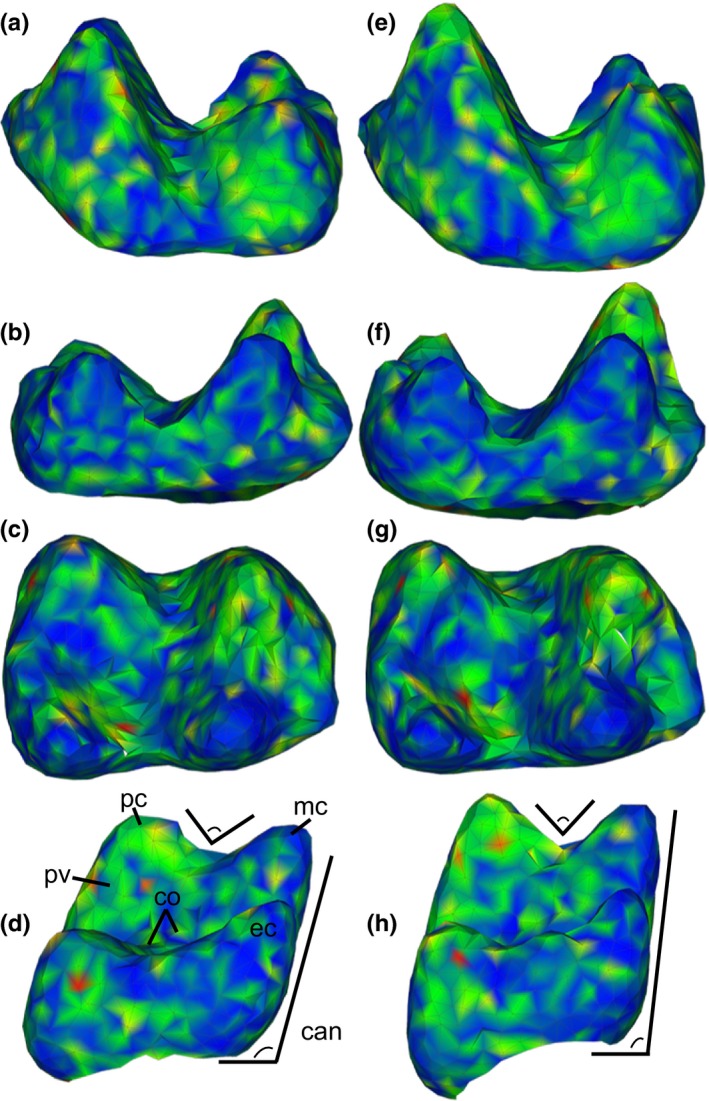
Shapes represented by the extremes of the first principal component (PC 1) of a dataset of two erinaceomorph taxa; (a) buccal, (b) lingual, (c) occlusal, and (d) posterior views of the minimum of PC 1; (e–h) same views of the maximum of PC 1. Shapes also generally correspond to shape differences between the two taxa, *Macrocranion junnei* (minimum) and cf. *Colpocherus* sp. (maximum) included in the analysis. Shapes are colored by the squared Euclidean distance between each point in the two surfaces. Warmer colors indicate greater distances. can, lingual canting angle; co, crista obliqua; ec, entoconid; mc, metaconid; pc, protoconid; pv, postvallid

**Figure 6:**
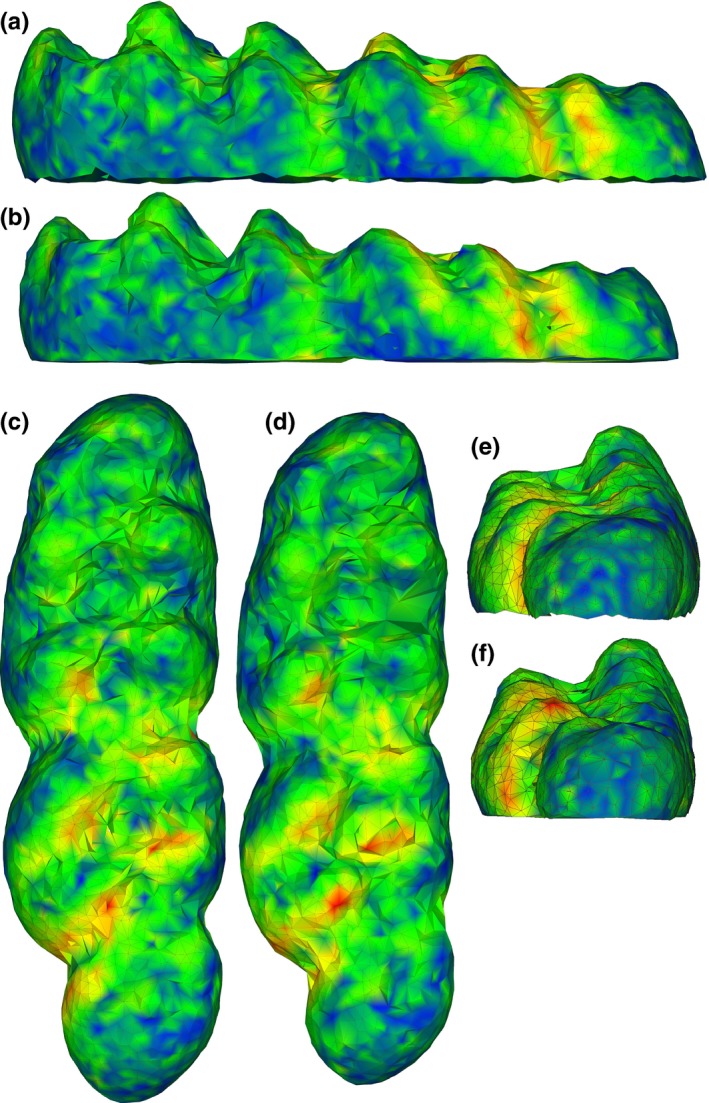
Mean shapes of the molar rows of the two experimental groups of *Mus musculus* analyzed by Gonzalez et al. (2016); (a–b) buccal, (c–d) dorsal, and (e–f) posterior view. Shapes are colored by the squared Euclidean distances between each point on the (a, c, e) control group mean shape and (b, d, f) growth hormone‐deficient mean shape. Warmer colors indicate greater distances

For each dataset, we chose the first replicate alignment from the best‐performing combination of pseudolandmark and mesh settings to visualize variation within the dataset. To explore whether primary patterns of variation in each dataset are related to taxonomy, tooth position, or some other factor, heat maps were generated describing differences along PC 1 (Figures 4 and 5) or between experimental groups (Figure 6). Color temperature in the maps was determined by the Euclidean distance between each pseudolandmark for two shapes calculated from a representative optimal alignment. Distances were squared to highlight patterns of difference on the tooth surface.

To assess the amount of error introduced by surface generation, three specimens were arbitrarily chosen for surface replication within the marsupial dataset (UF 325968, UF 330276, UF 332041). For each of those three specimens, a new, cropped surface file was created five times. Those fifteen replicate surfaces were added to the original dataset to create a fifth human error assessment dataset (*N* = 45). All surface files, including replicate surface files, were created by one of the authors (NSV). The human error assessment dataset was aligned three times, for a total of 60 alignment replicates. For each alignment, Procrustes ANOVA was computed and mean Euclidean distance between replicate surfaces and nonreplicate surfaces in principal component space was compared. The former procedure is a common tool for assessing measurement error (Fruciano, 2016; Klingenberg & McIntyre, 1998). The latter procedure is similar to previous assessments of segmentation error in CT‐scanned specimens (Boyer, 2008) as well as to assessments of overall digitization error in 2D geometric morphometric studies (von Cramon‐Taubadel, Frazier, & Lahr, 2007; Lockwood, Lynch, & Kimbel, 2002).

A suite of potentially discriminatory characters was derived for the heat maps of the erinaceomorph dataset. Each specimen was examined post hoc and scored for each character. Analyses of replicates were performed using R 3.0.2, including the packages geomorph (Adams & Otarola‐Castillo, 2013), plot3D (Soetaert, 2016), and plotrix (Lemon, 2006).

## 3 RESULTS

### 3.1 Simulation

**Figure 7:**
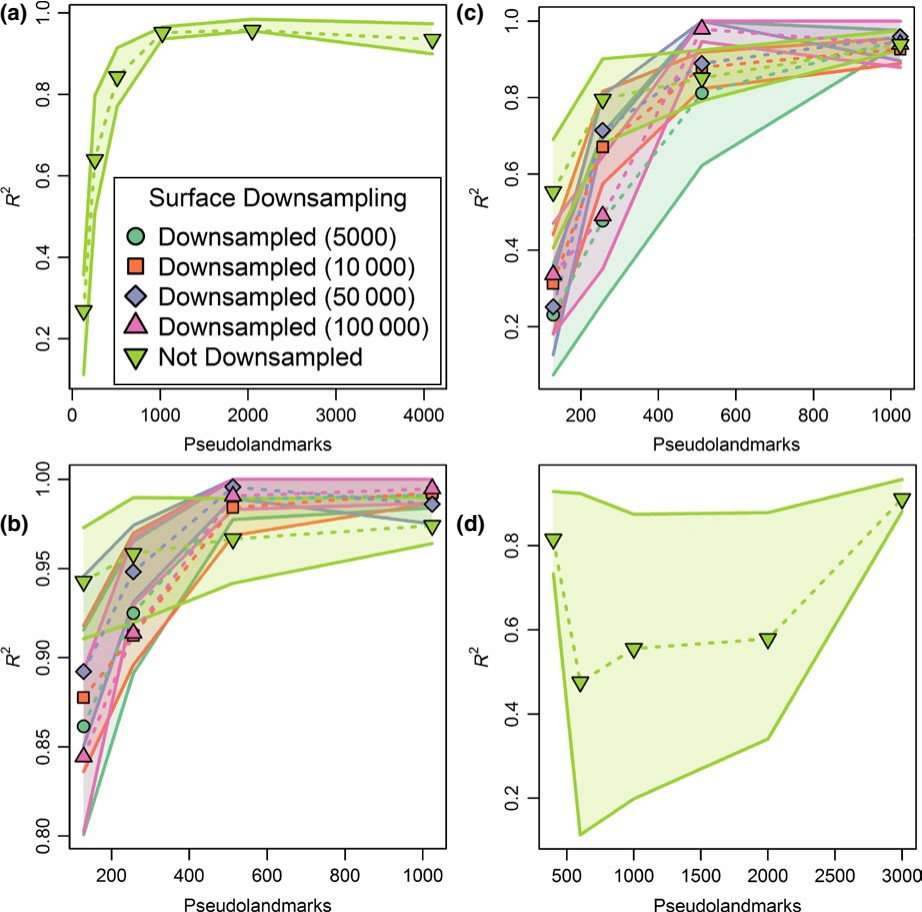
Plots of mean alignment repeatability (*R* ^2^ of replicate PC 1 values) for different combinations of pseudolandmarks used in alignment and downsampling settings of the input surfaces. Shaded regions around lines are interquartile ranges. Results are calculated from nine replicate alignments per combination and are plotted for (a) simulation, (b) marsupial, (c) erinaceomorph, and (d) *Mus musculus* datasets

**Figure 8:**
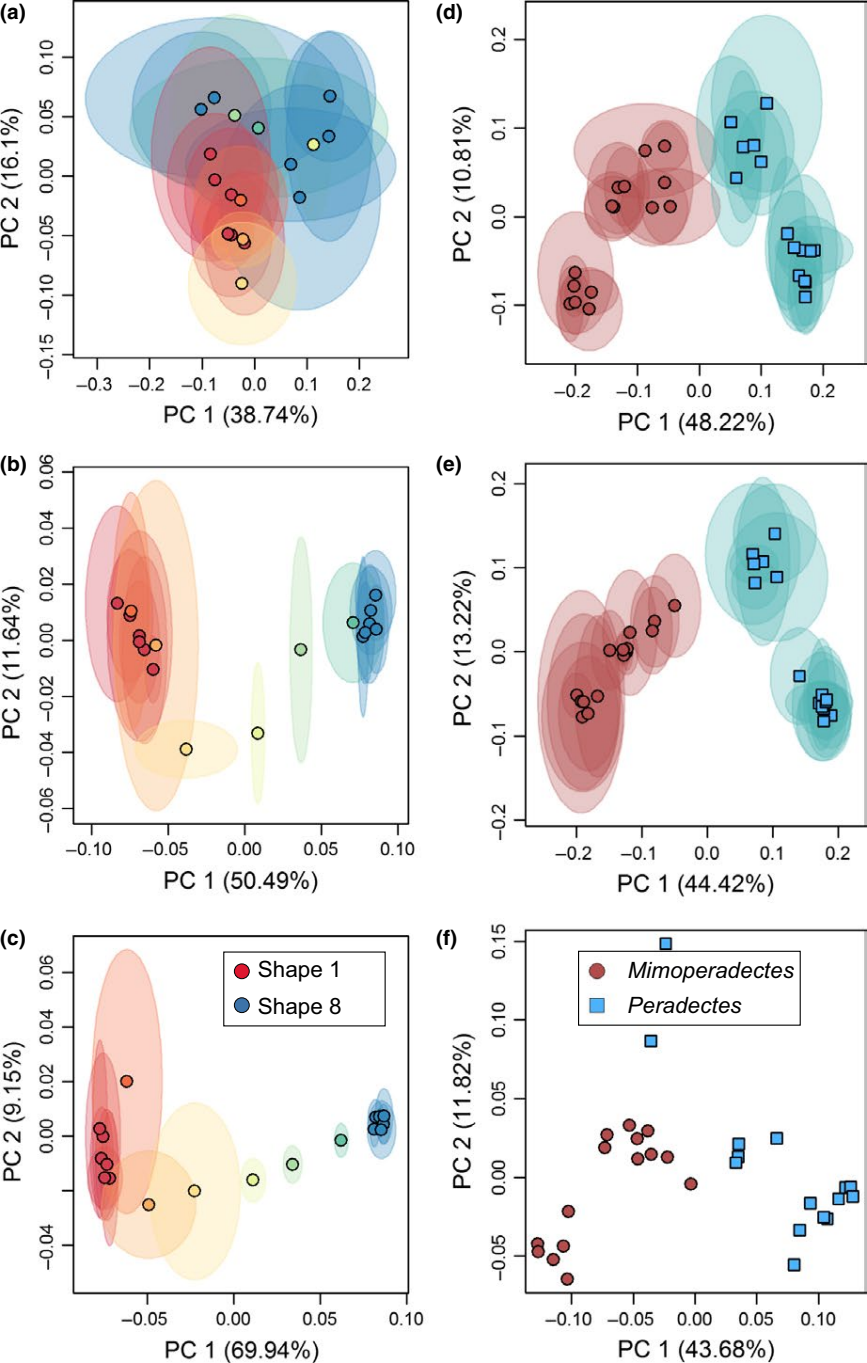
Principal components plots for (a–c) simulated and (d–f) marsupial datasets demonstrating the degree of alignment repeatability and separation between two taxa at different alignment settings. (a) 128‐point alignment, *R* ^2^ = 0.269; (b) 2048‐point alignment, *R* ^2^ = 0.96; (c) 4,096‐point alignment, *R* ^2^ = 0.94; (d) 5,000‐triangle surfaces at 128‐point alignment, *R* ^2^ = 0.231; (e), *R* ^2^ = 0.927; (f), *R* ^2^ = 0.960. Shaded regions around each point indicate median absolute deviation of location of each specimen in principal component space based on nine replicate alignments

**Figure 9:**
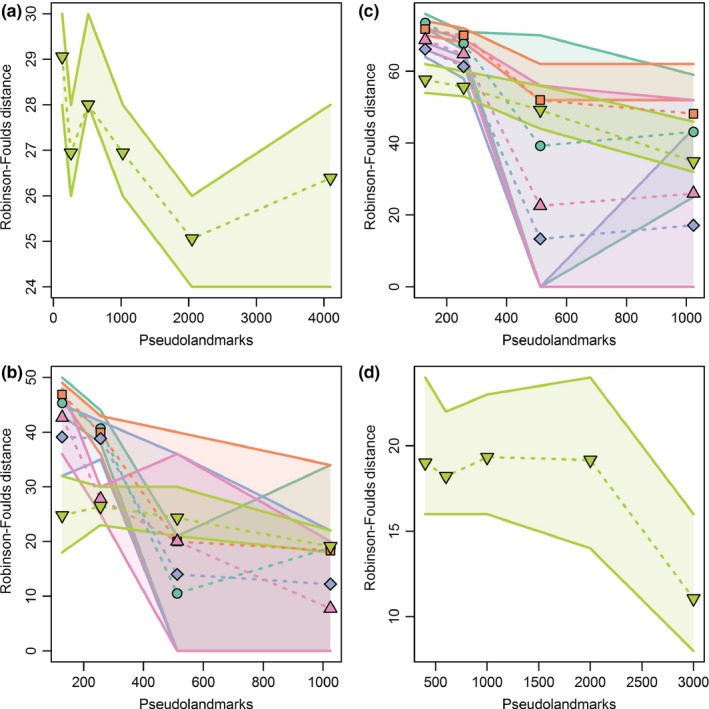
Plots of mean Robinson‐Foulds distances measuring differences between pairs of dendrograms resulting from cluster analyses of replicate alignments of specimens for different combinations of pseudolandmarks used in alignment and downsampling settings of the input surfaces. Shaded regions around lines are interquartile ranges. Results are calculated from nine replicate alignments per combination and are plotted for (a) simulation, (b) marsupial, (c) erinaceomorph, and (d) *Mus musculus* datasets. Colors and shapes correspond to the legend in Figure 7

Mean and median values were similar for correlation coefficients, *p*‐values, and Robinson‐Foulds distances for the simulated dataset (Supplementary Data). Only mean values are reported here. Correlations between PC 1 values of replicate alignments increased monotonically until 1,024‐point sampling, after which the correlation did not become significantly stronger (Figure 7a). On average, *R* ^2^ between PC 1 scores of replicated analyses was 0.27 for 128 pseudolandmarks, 0.64 for 256 pseudolandmarks, 0.84 for 512 pseudolandmarks, 0.95 for 1,024 pseudolandmarks, 0.96 for 2048 pseudolandmarks, and 0.94 for 4,096 pseudolandmarks. The two sets of replicated shapes overlapped each other in the first two principal components at 128‐point alignments (Figure 8a), but occupied separate regions of tangent shape space at all higher pseudolandmark settings. At progressively higher pseudolandmark settings, the space in the first two principal components occupied by identical shapes shrunk and overlapped with fewer intermediate shapes (Figure 8b,c). Mantel test *p*‐values were relatively high (0.43 < *p* < 0.48) for all pseudolandmark settings except for 4,096‐point sampling (Supplementary Data). Among those replicates with 4,096‐point sampling, the mean *p*‐value approached zero (*p* < 0.001). Robinson‐Foulds distances neither approached zero nor consistently decreased with increasing pseudolandmark values (25 < distance < 29; Figure 9a).

**Figure 10:**
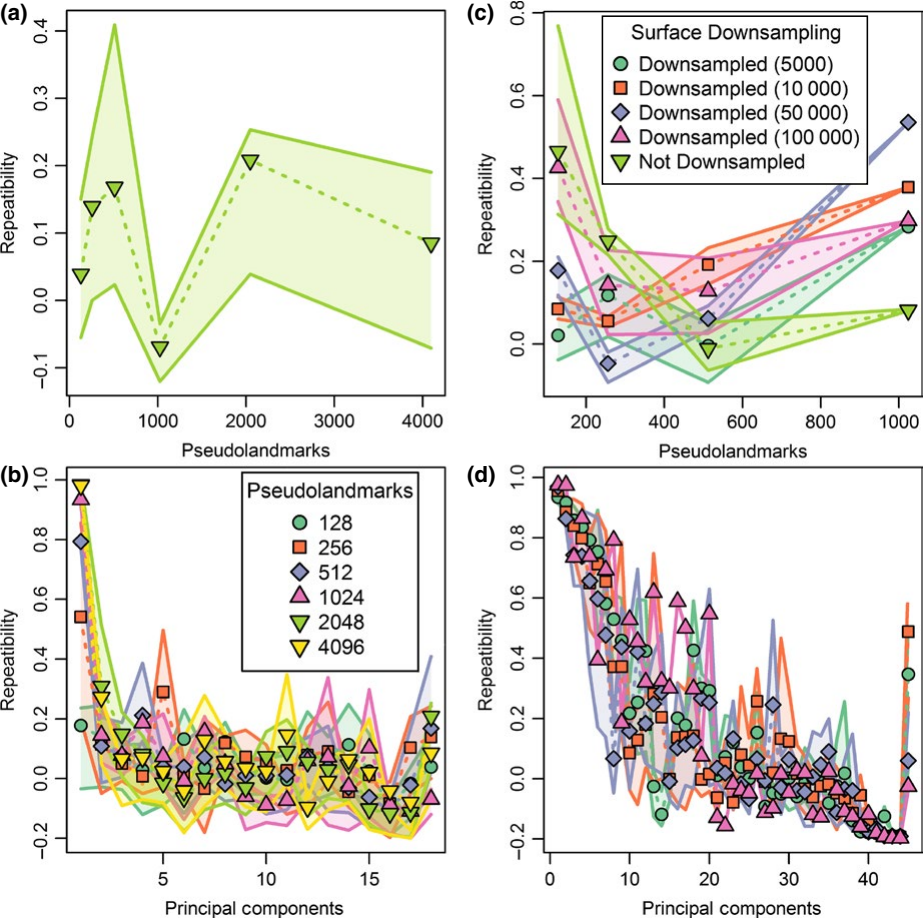
Plots of Procrustes ANOVA measures of repeatability in pseudolandmark placement (a, c) for all principal components together and (b, d) for each principal component individually for different combinations of pseudolandmarks used in alignment and downsampling settings of the input surfaces. (a–b) simulated dataset measuring error due to algorithmic pseudolandmark placement error (PPE) alone; (c–d) marsupial dataset downsampled to 100k surfaces, measuring error due to both PPE and human cropping of surfaces before alignment. Shaded regions around lines are interquartile ranges. Results are calculated from nine replicate alignments per combination. Upper legend corresponds to parts a, b. Lower legend corresponds to parts b, d

In contrast to the correspondence between increased pseudolandmark sampling and stronger correlations between PC 1 values of replicate alignments, Procrustes ANOVA repeatability values were low for the full dataset at all pseudolandmark settings (Figure 10a). To explore this inconsistency, repeatability was calculated for each PC separately. Mean repeatability values for PC1 were broadly consistent with correlation coefficients but low (repeatability <0.4) for all subsequent PCs at all pseudolandmark settings (Figure 10b).

### 3.2 Marsupials

The correlations of PC 1 values in terms of mean and median *R* ^2^ values were similar across datasets with different numbers of surface triangles and pseudolandmarks per specimen, differing on average by only 1%. Correlations were affected more by how many pseudolandmarks were used to align specimens than by how downsampled the surfaces were (Figure 7b). Results from mean values are presented first. On average, *R* ^2^ between PC 1 scores of replicated analyses was 0.83 for 128 pseudolandmarks, 0.93 for 256 pseudolandmarks, and 0.99 for 512 and 1,024 pseudolandmarks. At each level of pseudolandmarks, results from different downsampling settings were similar. At the full resolution setting, repeatability was high and the number of pseudolandmarks used did not make a significant difference in mean *R* ^2^ values. Across downsampling levels, 1,024‐point pseudolandmark alignments did not perform significantly better or worse than 512‐point pseudolandmark alignments, and at those settings, no downsampling level performed significantly better than any other. When median *R* ^2^ values were compared, specimens downsampled to 5k, 10k, and 100k faces all performed better than nondownsampled specimens at 1,024‐point alignment (*p* = 0.038, *p* = 0.038, *p* = 0.001, respectively), and specimens downsampled to 5k and 50k faces performed significantly better than nondownsampled faces at 512‐point alignment (*p* = 0.027, *p* = 0.0005, respectively; Supplementary Data).

Alignment settings with mean *R* ^2^ below 0.89 did not consistently separate the two taxa in the first three principal components (Figure 6d). Those with mean *R* ^2^ of 0.89–0.95 variably separated the two taxa (Figure 6e), and those with *R* ^2^ > 0.95 consistently separated the taxa (Figure 6f). Results of both Mantel tests and dendrogram comparisons were highly bimodal at high‐pseudolandmark settings, resulting in high standard deviations and large differences between mean and median values (Supplementary Data). Mean and median Mantel test *p*‐values approached zero above 100k faces and 512 pseudolandmarks. However, they were highly variable across comparison (0 < *p* < .999) and did not monotonically decrease as pseudolandmark values increased. In contrast, mean Robinson‐Foulds distances between pairs of dendrograms decreased as pseudolandmark values increased with the exception of surfaces downsampled to 5k faces (Figure 9b). For all downsampling levels, except for full resolution alignments, the greatest decrease in distance between trees was between 256‐point and 512‐point sampling with little further decrease in distance at 1,024‐point sampling. Mean distances did not reach zero for any combination of settings, but median Robinson‐Foulds distances were zero at 50k faces and 512‐point alignments, and 100k faces and 1,024‐point alignment (Supplementary Data).

Human surface generation error estimates were also affected by alignment parameters. Where repeatability was lowest, mean distance due to surface generation error was 2.2% of mean distance between specimens (100k‐triangle downsampling, 128‐point alignment). Where repeatability was highest, mean surface generation error was 0.8% of between‐specimen distance (100k‐triangle downsampling, 1,024‐point alignment). Highest mean surface generation error estimates were calculated from 128‐point alignments using 10k‐triangle downsampling (7.3% of between‐specimen distance). Lowest error estimates came from the same alignment settings that produced highest mean *R* ^2^ (Supplementary Data).

In contrast to error measured by distances, error measured by Procrustes ANOVA of the complete set of principal components was consistently high across all datasets (0 < mean Procrustes ANOVA repeatability <0.53; Figure 10c). Given that high levels of PPE resulted in low repeatability even for specimens that were identical (Figure 10a), we also measured repeatability for each PC for specimens downsampled to 100k faces (Figure 10d). For all pseudolandmark levels, mean repeatability along PC 1 was above 0.95 for most pseudolandmark settings (128 = 0.93, 256 = 0.95, 512 = 0.97, 1,024 = 0.98) but along PC 2 only 1,024‐point alignments had high mean repeatability (1,024 = 0.97).

Based on maximum *R* ^2^ in terms of both means and medians, as well as the usefulness of 1,024‐point alignments for reconstructing hypothetical surfaces, the first replicate using 100k triangles at 1,024‐point alignment was used to visualize shape variation. PC 1 described shape differences between the two taxa *Peradectes protinnominatus* and *Mimoperadectes labrus*, with the maximum values of PC 1 corresponding to *Peradectes protinnominatus* and the minimum values corresponding to *Mimoperadectes labrus* (Figure 3). The major differences between the two taxa, as represented by PC 1, include a wider trigonid and talonid basin relative to the length of each basin and a shallower hypoflexid (straighter cristid obliqua) in *P. protinnaminatus* compared to that of *M. labrus*. A wide talonid and shallow hypoflexid are noted as particularly indicative of Wa‐0 *P. protinnominatus* (“cf. *P. protinnominatus*” in Rose et al. (2012)). Additionally, in *M. labrus*, the metaconid and entoconid are shorter and blunter, while the protoconid and hypoconid are narrower and taller than in *P. protinnominutus*, although it is possible that the differences in cusp height may be affected by wear to the tooth crown.

### 3.3 Erinaceomorphs

For comparisons of PC 1 values, some distributions of *R* ^2^ were bimodal between more disparate values than in the marsupial dataset, causing greater differences between mean and median *R* ^2^. Mean differences between mean and median were 3% and had a maximum of 11% (50k faces sampled at 512 pseudolandmarks, mean *R* ^2^ = 0.88, median *R* ^2^ = 1).

*R* ^2^ values were generally lower for the erinaceomorph dataset than the marsupial dataset at a given parameter setting (Figure 7c). A line graph of median *R* ^2^ values has a similar pattern to Figure 7c and is not figured. Rather than converge near 1, at 1,024‐point pseudolandmark sampling mean *R* ^2^ values converged on 0.93–0.96. *R* ^2^ values were also more widely distributed, resulting in larger confidence intervals. When comparing means using Bonferroni's‐corrected pairwise *t*‐tests, surfaces sampled with at least 10k triangles and with at least 512 pseudolandmarks produced alignments that had statistically insignificant differences between *R* ^2^ values assessed at *p* = 0.05. In fact, most values were at or near *p* = 1 with the exception of the comparison of 100k‐triangle downsampled and not downsampled datasets at 512 pseudolandmark points, where surfaces with 100k faces performed slightly better than surfaces with full resolution (means: 0.85 vs. 0.97; Bonferroni‐corrected *p* = 0.063). When comparing medians using Bonferroni‐corrected Mann–Whitney *U*‐tests, results were similar with some exceptions. Fifty thousand triangle and 100k triangle downsampled datasets aligned using 512 points performed significantly better than other datasets aligned at 512 points (0 < *p* < 0.02). No 1,024‐point alignment performed better or worse than its matching 512‐point alignment except in the case of full resolution, where increasing the number of pseudolandmarks improved performance. No resolution setting performed better than any other at 1,024‐point alignment.

Mean and median *p*‐values resulting from Mantel tests were generally similar and did not decrease with increasing pseudolandmark values. *p*‐values ranged widely (0 < *p* < 0.999), but means were generally high (0.13 < *p* < 0.42), with two exceptions: values for 50k faces and 512 and 1,024‐point alignments both approached zero (Supplementary Data).

Distances between dendrograms were highly bimodal at high‐pseudolandmark settings, resulting in high standard deviations and large differences between mean and median values (Figure 9c). Mean Robinson‐Foulds distances between pairs of dendrograms decreased as pseudolandmark values increased from 256‐point to 512‐point sampling, but did not decrease further with increased pseudolandmark sampling. Mean distances did not reach zero for any combination of settings, but median Robinson‐Foulds distances were zero at 50k faces and 512‐point alignments, 50k faces and 1,024‐point alignments, and 100k faces and 512‐point alignment (Supplementary Data).

Based on maximum *R* ^2^ in terms of both means and medians, as well as the usefulness of 1,024‐point alignments for reconstructing hypothetical surfaces, the first replicate using 50k triangles at 1,024 point alignment was used to visualize shape variation. Maximum and minimum shapes along PC 1 differed in that the minimum shape had (i) a greater degree of lingual canting, (ii) a more obtuse angle between the protoconid and metaconid above the postvallid, (iii) a more medially reaching cristid obliqua that partially climbed the postvallid toward the metaconid, and (iv) a more triangular and wider entoconid than the narrow and curved entoconid of the maximum shape (Figure 4). Those same features differentiated the two specimens identified a priori as cf. *Colpocherus* sp. and *Macrocranion junnei*, and the greater degree of lingual canting in *M. junnei* has been noted as a possible distinguishing feature between the two species (Rose et al., 2012). Crown surfaces of all specimens were subsequently examined in Meshlab and scored for each of the four characters described above. All specimens matched the character scoring of either extreme of PC 1. No specimen had a “mixed” character scoring combining features of both extremes. Those results were used to identify all specimens in the analysis a posteriori as either cf. *Colpocherus* sp. (the maximum shape along PC 1) or *Macrocranion junnei* (the minimum PC 1 shape). Other features that are proposed to differentiate between *M. junnei* and cf. *Colpocherus* sp. include a taller trigonid, protoconid, and hypoconid in cf. *Colpocherus* sp. (Rose et al., 2012). These features are also apparent in the maximum shape as compared to the minimum shape of PC 1, but they may also be affected by wear to the tooth crown.

**Figure 11:**
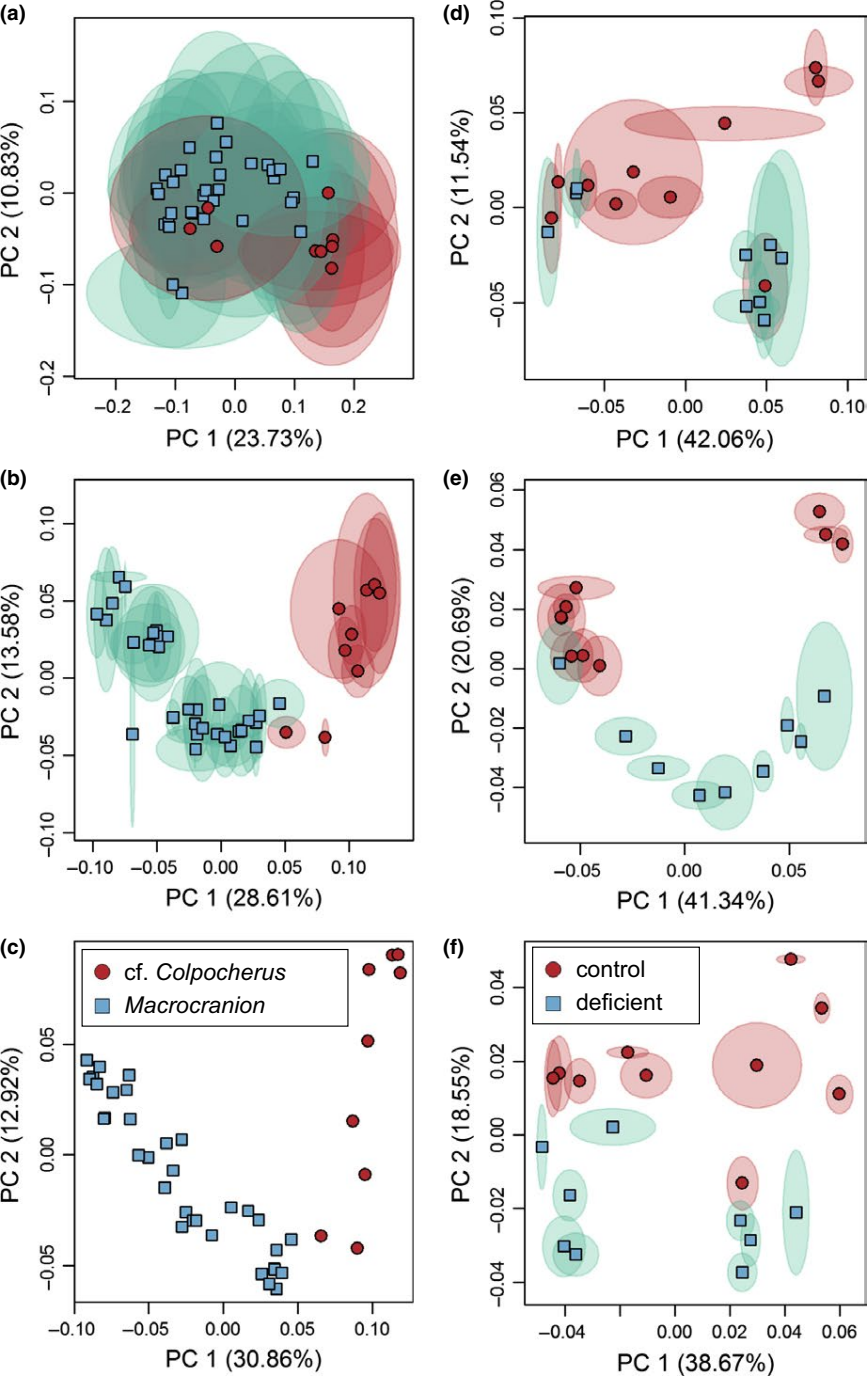
Principal components plots for (a–c) erinaceomorph and (d–f) *Mus musculus* datasets demonstrating the degree of alignment repeatability and separation between two taxa at different alignment settings. (a) 5,000‐triangle surfaces at 128‐point alignment, *R* ^2^ = 0.231; (b) 10,000‐triangle surfaces at 1,024‐point alignment, *R* ^2^ = 0.927; (c) 50,000‐triangle surfaces at 1,024‐point alignment, *R* ^2^ = 0.960; (d) 399‐point alignment, *R* ^2^ = 0.815; (e) 1,000‐point alignment, *R* ^2^ = 0.555; (f) 3,000‐point alignment, *R* ^2^ = 0.911. Shaded regions around each point indicate median absolute deviation of location of each specimen in principal component space based on nine replicate alignments

Alignment settings with mean *R* ^2^ below 0.81 did not consistently separate the two taxa in the first three principal components (Figure 11a). Those with mean *R* ^2^ 0.81–0.94 variably separated the two taxa (Figure 11b), and those with *R* ^2^ > 0.94 consistently separated the taxa (Figure 11c).

### 3.4 Mus musculus

The *Mus musculus* sample showed the same overall trend of increasing repeatability with increasing number of pseudolandmarks that was observed in other datasets (Figure 7d). *R* ^2^ values were unusually high for 399‐point alignments. *R* ^2^ values were generally lower for the mouse molar row dataset than the marsupial and erinaceomorph datasets at similar parameter settings. Even when the per‐tooth number of pseudolandmarks was similar between datasets (3,000 pseudolandmarks for three‐tooth mouse molar row, 1,024 pseudolandmarks per marsupial or erinaceomorph molar), repeatability was lower for the mouse molars (mean *R* ^2^ = 0.91) than for the marsupial or erinaceomorph molars (mean *R* ^2^ = 0.93–0.99). Mean and median Mantel test *p*‐values were uniformly low across pseudolandmark settings (*p* < 0.085; Supplementary Data). Similar to other datasets, Robinson‐Foulds distances between dendrograms generally decreased as the number of pseudolandmarks used in alignments increased, but mean and median distances never reached zero. The greatest decrease in mean distance occurred between 2,000 and 3,000‐point alignments (Figure 9d).

Using the same 399‐point configuration, our alignments produced principal components scatters similar to Gonzalez et al. (2016: figure 3b, compared to Figure 11d). When higher pseudolandmark numbers were used to align specimens, separation between the two experimental groups was comparable to the results produced from use of landmarks, contours, and semilandmarks (Figure 11e,f compare to Gonzalez et al., 2016: figure 2). The first replicate using 3,000‐point alignment was used to visualize differences between the two experimental groups (Figure 6). Differences were strongest on the hypoconid of M/1, the M/2, and the buccoanterior region of M/3.

## 4 DISCUSSION

Our results support the utility of auto3dgm for characterizing morphological differences between highly similar or pseudocryptic species, and even between different experimental strains of the same species (contra results of Gonzalez et al., 2016). Analyses of alignments produced by an adequately high number of pseudolandmarks produce results comparable to those produced by other geometric morphometric methods (Figure 11f, compare to Gonzalez et al., 2016: figure 2, Boyer et al., 2015a). We found that choice of alignment settings, particularly pseudolandmark settings, makes a significant difference in the performance of auto3dgm. Our results show that not all alignment settings perform equally well, and also that identical settings do not perform equally well with different datasets. Importantly, all alignments in our dataset contained a nontrivial amount of PPE, as seen in the low Procrustes ANOVA repeatability scores of the simulated and marsupial human error datasets as well as the nonzero Robinson‐Foulds distances when comparing replicate alignments. In particular, multiple analyses of the simulated dataset resulted in low similarity between alignments when that entire dataset was analyzed in large part because the placement of two‐thirds of the simulated dataset in tangent shape space was entirely determined by PPE (replicate Shapes 1 and 8, Figure 8d–f). Nonzero distances between dendrograms constructed from replicate alignments and low repeatability values for the complete simulated dataset support the conclusion that even when PPE is not greater than the true pattern of variation in a dataset, it still comprises a nontrivial proportion of total variation in an alignment.

These results in total strongly support the suggestion that sample‐specific sensitivity analyses should be performed as part of the protocol for auto3dgm in order to determine what settings are adequate for a given sample. We recommend that users include multiple identical copies of at least one shape in their alignments, then measure the Procrustes ANOVA repeatability score for each principal component in turn. Further analyses should be limited to only those PCs with repeatability scores above a given threshold. That protocol is similar to those used as part of assessing user‐placement error in landmark‐based geometric morphometric datasets (Fruciano, 2016). Alternatively, the entire dataset could be repeatedly aligned and correlations between corresponding principal components could be measured. Based on our results, correlation coefficients of 0.94 or higher indicate reliable alignment settings. Results from median reproducibility values also support the suggestion that standardization of face number across the dataset may improve alignment results, as long as the standardized triangle value is high enough.

In the case of the two datasets composed of relatively simple, isolated fossil molars examined here, alignments using at least 512 pseudolandmarks and surfaces composed of at least 50k triangles adequately separated the two taxa included in each analysis. At optimal pseudolandmark settings, surfaces standardized to a high number of faces (50k or 100k triangles) slightly outperformed the raw, full‐resolution surfaces. Differences in surface downsampling settings affected reproducibility less than differences in pseudolandmark settings.

Based on our reanalysis of data from the study by Gonzalez et al. (2016), we conclude that parameterization choices contributed to the relatively poor automated results previously obtained for teeth of *M. musculus*. Those authors used mesh models with a low number of faces ranging from 1,500 to 6,500 triangles. Such low‐resolution input surfaces could potentially lead to suboptimal configurations of landmarks in the initial steps of the automated procedure. We were unable to test for the effects of resolution on the results because the face numbers reported above represent the full‐resolution faces. Further downsampling of those original meshes would have been unlikely to improve results. Therefore, our results also use the original mesh models analyzed by Gonzalez et al. (2016). More importantly, Gonzalez et al. (2016) used a maximum of 1,000 pseudolandmarks to characterize their shapes, which is likely to cause problems because that number is too sparse to overcome PPE in the face of localized differences along the complex shape of the molar rows that they analyzed. When we used higher numbers of pseudolandmarks, results improved and produced results similar to those produced by placement of 3D landmarks and sliding semilandmarks (Gonzalez et al., 2016). Based on the steep increase in PC 1 correlation between 2000 and 3,000‐point alignments (Figure 7), we suspect that use of an even higher number of pseudolandmarks than the maximum used in this study would be optimal for the *Mus musculus* dataset of Gonzalez et al. (2016).

Our results support the hypothesis from previous studies that adequate alignment settings will also be affected by the degree of variation between shapes (Gonzalez et al., 2016). With increased variability between shapes, fewer pseudolandmarks are needed to outweigh the error introduced by random landmark placement and to reliably characterize variation in the dataset. For example, a dataset of 116 second lower molars sampled from across a spectrum of small eutherian mammals was aligned multiple times in a previous pilot study (Boyer et al., 2015a). Similar to the results of our study, repeatability (*R* ^2^) was high: 0.85 for 128‐point alignments, 0.92 for 200‐point alignments, and 0.95 for 1,000‐point alignments. In contrast, with a dataset like that of Gonzalez et al. (2016), in which molar rows with high overall similarity were being compared between two strains of the same species, more than an order of magnitude more pseudolandmarks than were used in the original analysis of specimens were needed to adequately describe the variation in that dataset.

Overall, we find that the performance of auto3dgm is adequate for studying samples in which the variation of interest is relatively subtle, such as when the ratio of intergroup to intragroup variation is small, provided that the sampling density of pseudolandmarks is adequate to overcome the random error introduced by PPE and the resolution of the mesh is substantially greater than the number of pseudolandmarks used. Therefore, auto3dgm is a promising tool for use in characterizing morphological systems that have remained stubbornly difficult to separate (Carrasco, 1998; Sáez et al., 2003) and to determine the patterns and limits of morphological variation, whether it be within species, between species, or between higher clades.

## CONFLICT OF INTEREST

The authors declare no conflict of interests.
