# Supplementary material for: Semi‐supervised determination of pseudocryptic morphotypes using observer‐free characterizations of anatomical alignment and shape
Source: Ecol Evol. 2017 Jun 2;7(14):5041–55. doi: 10.1002/ece3.3058 (PMC5528226; doi:10.1002/ece3.3058)
Supplement: Supplementary file 1 [file ECE3-7-5041-s001.docx]

**Supplementary Data: Semi-supervised determination of pseudocryptic morphotypes using observer-free characterizations of anatomical alignment and shape**

**Table 1.** Summary statistics for correlation coefficients (R^2^) between PC1 values in replicated alignments of the simulated dataset.

| **Triangles per Mesh** | **Pseudolandmarks** | **Mean** | **Median** | **Standard Deviation** | **Minimum** | **Maximum** |
| --- | --- | --- | --- | --- | --- | --- |
| **8,192** | **128** | 0.269 | 0.223 | 0.211 | 0.004 | 0.809 |
| **8,192** | **256** | 0.64 | 0.65 | 0.199 | 0.191 | 0.928 |
| **8,192** | **512** | 0.843 | 0.869 | 0.089 | 0.597 | 0.969 |
| **8,192** | **1,024** | 0.952 | 0.954 | 0.021 | 0.909 | 0.987 |
| **8,192** | **2,048** | 0.957 | 0.972 | 0.04 | 0.846 | 0.994 |
| **8,192** | **4,096** | 0.935 | 0.946 | 0.042 | 0.854 | 0.99 |

**Table 2.** Bonferroni-corrected p-values for tests of significant differences between mean correlation coefficients (R^2^) of between PC1 values in replicated alignments of the simulated dataset. Abbreviations: P = pseudolandmarks.

|  | **128P** | **256P** | **512P** | **1024P** | **2048P** |
| --- | --- | --- | --- | --- | --- |
| **256P** | 0 |  |  |  |  |
| **512P** | 0 | 0 |  |  |  |
| **1024P** | 0 | 0 | 0.005 |  |  |
| **2048P** | 0 | 0 | 0.003 | 1 |  |
| **4096P** | 0 | 0 | 0.035 | 1 | 1 |

**Table 3.** Summary statistics for Mantel tests (p-values) comparing replicated alignments of simulated dataset.

| **Pseudolandmarks** | **Value** | **Mean** | **Median** | **Standard Deviation** | **Minimum** | **Maximum** |
| --- | --- | --- | --- | --- | --- | --- |
| **128** | **R** | 0.489 | 0.438 | 0.273 | 0.038 | 0.957 |
| **256** | **R** | 0.432 | 0.343 | 0.279 | 0 | 0.943 |
| **512** | **R** | 0.438 | 0.312 | 0.305 | 0 | 0.998 |
| **1,024** | **R** | 0.458 | 0.418 | 0.314 | 0.002 | 0.956 |
| **2,048** | **R** | 0.45 | 0.448 | 0.315 | 0 | 0.988 |
| **4,096** | **R** | 0.001 | 0 | 0.003 | 0 | 0.014 |

**Table 4.** Summary statistics for Robinson-Foulds distances between pairs of phenograms constructed from replicated alignments of the simulated dataset.

| **Triangles per Mesh** | **Pseudolandmarks** | **Mean** | **Median** | **Standard Deviation** | **Minimum** | **Maximum** |
| --- | --- | --- | --- | --- | --- | --- |
| **8,192** | **128** | 29.056 | 30 | 1.393 | 26 | 30 |
| **8,192** | **256** | 26.944 | 28 | 2.216 | 20 | 30 |
| **8,192** | **512** | 28 | 28 | 1.912 | 20 | 30 |
| **8,192** | **1,024** | 26.944 | 28 | 2.216 | 22 | 30 |
| **8,192** | **2,048** | 25.056 | 26 | 2.808 | 18 | 30 |
| **8,192** | **4,096** | 26.389 | 26 | 2.333 | 22 | 30 |

**Table 5.** Summary statistics for correlation coefficients (R^2^) between PC1 values in replicated alignments of the marsupial dataset.

| **Triangles per Mesh** | **Pseudolandmarks** | **Mean** | **Median** | **Standard Deviation** | **Minimum** | **Maximum** |
| --- | --- | --- | --- | --- | --- | --- |
| **5,000** | **128** | 0.862 | 0.861 | 0.071 | 0.727 | 1 |
| **5,000** | **256** | 0.925 | 0.933 | 0.045 | 0.838 | 0.983 |
| **5,000** | **512** | 0.989 | 0.989 | 0.011 | 0.978 | 1 |
| **5,000** | **1,024** | 0.991 | 0.984 | 0.008 | 0.984 | 1 |
| **10,000** | **128** | 0.878 | 0.876 | 0.054 | 0.78 | 0.978 |
| **10,000** | **256** | 0.913 | 0.954 | 0.087 | 0.679 | 0.986 |
| **10,000** | **512** | 0.984 | 0.984 | 0.016 | 0.968 | 1 |
| **10,000** | **1,024** | 0.993 | 0.987 | 0.007 | 0.987 | 1 |
| **50,000** | **128** | 0.892 | 0.915 | 0.07 | 0.669 | 0.969 |
| **50,000** | **256** | 0.948 | 0.953 | 0.03 | 0.865 | 0.984 |
| **50,000** | **512** | 0.996 | 1 | 0.005 | 0.989 | 1 |
| **50,000** | **1,024** | 0.986 | 0.975 | 0.013 | 0.975 | 1 |
| **100,000** | **128** | 0.844 | 0.856 | 0.075 | 0.648 | 0.956 |
| **100,000** | **256** | 0.914 | 0.953 | 0.092 | 0.677 | 0.992 |
| **100,000** | **512** | 0.991 | 0.983 | 0.008 | 0.983 | 1 |
| **100,000** | **1,024** | 0.995 | 1 | 0.007 | 0.987 | 1 |
| **full resolution** | **128** | 0.943 | 0.945 | 0.039 | 0.857 | 0.998 |
| **full resolution** | **256** | 0.862 | 0.861 | 0.071 | 0.727 | 1 |
| **full resolution** | **512** | 0.925 | 0.933 | 0.045 | 0.838 | 0.983 |
| **full resolution** | **1,024** | 0.989 | 0.989 | 0.011 | 0.978 | 1 |

**Table 6.** Bonferroni-corrected p -values for tests of significant differences between mean correlation coefficients (R^2^) of between PC1 values in replicated alignments of the marsupial dataset. Abbreviations: FR = Full Resolution, k = thousand, P = pseudolandmarks, T = triangles per mesh.

|  | **5kT**  **128P** | **5kT**  **256P** | **5kT**  **512P** | **5kT**  **1024P** | **10k**  **T128P** | **10kT**  **256P** | **10kT**  **512P** | **10kT**  **1024P** | **50kT**  **128P** | **50kT**  **256P** |
| --- | --- | --- | --- | --- | --- | --- | --- | --- | --- | --- |
| **5kT256P** | 0 |  |  |  |  |  |  |  |  |  |
| **5kT512P** | 0 | 0 |  |  |  |  |  |  |  |  |
| **5kT1024P** | 0 | 0 | 0.018 |  |  |  |  |  |  |  |
| **10kT128P** | 1 | 0.001 | 0 | 0 |  |  |  |  |  |  |
| **10kT256P** | 0 | 0 | 0.015 | 0 | 0 |  |  |  |  |  |
| **10kT512P** | 0 | 0 | 1 | 1 | 0 | 0 |  |  |  |  |
| **10kT1024P** | 0 | 0 | 0.246 | 1 | 0 | 0 | 1 |  |  |  |
| **50kT128P** | 1 | 0 | 0 | 0 | 1 | 0 | 0 | 0 |  |  |
| **50kT256P** | 0 | 0 | 1 | 0 | 0 | 1 | 0.001 | 0 | 0 |  |
| **50kT512P** | 0 | 0 | 1 | 1 | 0 | 0 | 1 | 1 | 0 | 0 |
| **50kT1024P** | 0 | 0 | 0.007 | 1 | 0 | 0 | 1 | 1 | 0 | 0 |
| **100kT128P** | 0.633 | 0.014 | 0 | 0 | 1 | 0 | 0 | 0 | 1 | 0 |
| **100kT256P** | 0 | 1 | 0 | 0 | 0 | 0 | 0 | 0 | 0 | 0 |
| **100kT512P** | 0 | 0 | 0.001 | 1 | 0 | 0 | 0.975 | 1 | 0 | 0 |
| **100kT1024P** | 0 | 0 | 0.068 | 1 | 0 | 0 | 1 | 1 | 0 | 0 |
| **FRT128P** | 0 | 1 | 0 | 0 | 0 | 0.188 | 0 | 0 | 0 | 0.001 |
| **FRT256P** | 0 | 0 | 1 | 0.002 | 0 | 0.092 | 1 | 0.045 | 0 | 1 |
| **FRT512P** | 0 | 0 | 1 | 0.916 | 0 | 0 | 1 | 1 | 0 | 0.025 |
| **FRT1024P** | 0 | 0 | 0.07 | 1 | 0 | 0 | 1 | 1 | 0 | 0 |

**Table 6.** continued.

|  | **50kT**  **512P** | **50kT**  **1024P** | **100kT128P** | **100kT256P** | **100kT512P** | **100kT1024P** | **FRT128P** | **FRT**  **256P** | **FRT**  **512P** |
| --- | --- | --- | --- | --- | --- | --- | --- | --- | --- |
| **50kT1024P** | 1 |  |  |  |  |  |  |  |  |
| **100kT128P** | 0 | 0 |  |  |  |  |  |  |  |
| **100kT256P** | 0 | 0 | 0 |  |  |  |  |  |  |
| **100kT512P** | 1 | 1 | 0 | 0 |  |  |  |  |  |
| **100kT1024P** | 1 | 1 | 0 | 0 | 1 |  |  |  |  |
| **FRT128P** | 0 | 0.014 | 0 | 1 | 0.002 | 0 |  |  |  |
| **FRT256P** | 0.114 | 1 | 0 | 0.009 | 0.58 | 0.159 | 1 |  |  |
| **FRT512P** | 1 | 1 | 0 | 0 | 1 | 1 | 1 | 1 |  |
| **FRT1024P** | 1 | 1 | 0 | 0 | 1 | 1 | 0.744 | 1 | 1 |

**Table 7.** Summary statistics for Mantel tests (p-values) comparing replicated alignments of marsupial dataset.

| **Triangles per Mesh** | **Pseudolandmarks** | **Mean** | **Median** | **Standard Deviation** | **Minimum** | **Maximum** |
| --- | --- | --- | --- | --- | --- | --- |
| **5,000** | **128** | 0.201 | 0.012 | 0.299 | 0 | 0.999 |
| **5,000** | **256** | 0 | 0 | 0 | 0 | 0 |
| **5,000** | **512** | 0.356 | 0.331 | 0.362 | 0 | 0.773 |
| **5,000** | **1024** | 0.224 | 0.384 | 0.204 | 0 | 0.438 |
| **10,000** | **128** | 0.091 | 0 | 0.18 | 0 | 0.87 |
| **10,000** | **256** | 0.164 | 0.001 | 0.23 | 0 | 0.75 |
| **10,000** | **512** | 0.025 | 0.018 | 0.026 | 0 | 0.072 |
| **10,000** | **1024** | 0 | 0 | 0 | 0 | 0 |
| **50,000** | **128** | 0.188 | 0.063 | 0.264 | 0 | 0.915 |
| **50,000** | **256** | 0.189 | 0.071 | 0.237 | 0 | 0.73 |
| **50,000** | **512** | 0.262 | 0 | 0.333 | 0 | 0.703 |
| **50,000** | **1024** | 0.429 | 0.688 | 0.391 | 0 | 0.83 |
| **100,000** | **128** | 0.048 | 0 | 0.11 | 0 | 0.416 |
| **100,000** | **256** | 0.193 | 0.065 | 0.264 | 0 | 0.838 |
| **100,000** | **512** | 0 | 0 | 0 | 0 | 0.001 |
| **100,000** | **1024** | 0 | 0 | 0 | 0 | 0 |
| **full resolution** | **128** | 0 | 0 | 0 | 0 | 0 |
| **full resolution** | **256** | 0 | 0 | 0 | 0 | 0 |
| **full resolution** | **512** | 0 | 0 | 0 | 0 | 0 |
| **full resolution** | **1024** | 0 | 0 | 0 | 0 | 0 |

**Table 8.** Summary statistics for Robinson-Foulds distances between pairs of phenograms constructed from replicated alignments of the marsupial dataset.

| **Triangles per Mesh** | **Pseudolandmarks** | **Mean** | **Median** | **Standard Deviation** | **Minimum** | **Maximum** |
| --- | --- | --- | --- | --- | --- | --- |
| **5,000** | **128** | 45.333 | 46 | 8.919 | 0 | 52 |
| **5,000** | **256** | 40.667 | 40 | 4.356 | 30 | 52 |
| **5,000** | **512** | 10.5 | 10 | 10.673 | 0 | 22 |
| **5,000** | **1024** | 18.889 | 34 | 17.134 | 0 | 34 |
| **10,000** | **128** | 46.889 | 47 | 3.396 | 34 | 54 |
| **10,000** | **256** | 39.889 | 40 | 4.634 | 28 | 50 |
| **10,000** | **512** | 20 | 20 | 20.284 | 0 | 40 |
| **10,000** | **1024** | 18.333 | 32 | 16.648 | 0 | 34 |
| **50,000** | **128** | 39.111 | 42 | 7.178 | 24 | 50 |
| **50,000** | **256** | 38.833 | 40 | 5.417 | 28 | 50 |
| **50,000** | **512** | 14 | 0 | 17.799 | 0 | 36 |
| **50,000** | **1024** | 12.222 | 22 | 11.087 | 0 | 22 |
| **100,000** | **128** | 42.667 | 44 | 6.829 | 28 | 54 |
| **100,000** | **256** | 27.778 | 28 | 5.232 | 16 | 42 |
| **100,000** | **512** | 20 | 36 | 18.142 | 0 | 36 |
| **100,000** | **1024** | 7.778 | 0 | 9.888 | 0 | 20 |
| **full resolution** | **128** | 24.778 | 28 | 9.15 | 6 | 42 |
| **full resolution** | **256** | 45.333 | 46 | 8.919 | 0 | 52 |
| **full resolution** | **512** | 40.667 | 40 | 4.356 | 30 | 52 |
| **full resolution** | **1024** | 10.5 | 10 | 10.673 | 0 | 22 |

**Table 9.** Summary statistics for Euclidean distance-based measures of alignment error due to human cropping of surfaces in the marsupial dataset. Error between replicate surfaces was measured from five repeated crops of three specimens (UF 325968, UF 330276, UF 332041).

| **Triangles per Mesh** | **Pseudolandmarks** | **Non-replicate Mean** | **Non-replicate Standard Deviation** | **Replicate Mean** | **Replicate Standard Deviation** | **Ratio of Replicate to Non-replicate Mean** |
| --- | --- | --- | --- | --- | --- | --- |
| **5,000** | **128** | 0.37 | 0.097 | 0.024 | 0.016 | 0.064 |
| **5,000** | **256** | 0.3 | 0.083 | 0.016 | 0.011 | 0.052 |
| **5,000** | **512** | 0.221 | 0.055 | 0.007 | 0.004 | 0.029 |
| **5,000** | **1024** | 0.2 | 0.058 | 0.003 | 0.002 | 0.016 |
| **10,000** | **128** | 0.371 | 0.099 | 0.028 | 0.019 | 0.073 |
| **10,000** | **256** | 0.282 | 0.072 | 0.017 | 0.011 | 0.062 |
| **10,000** | **512** | 0.236 | 0.067 | 0.008 | 0.006 | 0.033 |
| **10,000** | **1024** | 0.19 | 0.053 | 0.007 | 0.006 | 0.035 |
| **50,000** | **128** | 0.349 | 0.089 | 0.007 | 0.005 | 0.021 |
| **50,000** | **256** | 0.288 | 0.077 | 0.01 | 0.007 | 0.035 |
| **50,000** | **512** | 0.226 | 0.06 | 0.004 | 0.003 | 0.02 |
| **50,000** | **1024** | 0.187 | 0.051 | 0.004 | 0.003 | 0.021 |
| **100,000** | **128** | 0.341 | 0.079 | 0.008 | 0.005 | 0.022 |
| **100,000** | **256** | 0.273 | 0.068 | 0.015 | 0.01 | 0.055 |
| **100,000** | **512** | 0.221 | 0.059 | 0.007 | 0.005 | 0.033 |
| **100,000** | **1024** | 0.196 | 0.053 | 0.002 | 0.001 | 0.009 |
| **full resolution** | **128** | 0.333 | 0.082 | 0.008 | 0.005 | 0.024 |
| **full resolution** | **256** | 0.274 | 0.068 | 0.004 | 0.003 | 0.016 |
| **full resolution** | **512** | 0.219 | 0.057 | 0.005 | 0.003 | 0.021 |
| **full resolution** | **1024** | 0.193 | 0.054 | 0.005 | 0.003 | 0.024 |

**Table 10.** Summary statistics for correlation coefficients (R^2^) between PC1 values in replicated alignments of the erinaceomorph dataset.

| **Triangles per Mesh** | **Pseudolandmarks** | **Mean** | **Median** | **Standard Deviation** | **Minimum** | **Maximum** |
| --- | --- | --- | --- | --- | --- | --- |
| **5,000** | **128** | 0.231 | 0.154 | 0.191 | 0.009 | 0.624 |
| **5,000** | **256** | 0.478 | 0.501 | 0.262 | 0.031 | 0.862 |
| **5,000** | **512** | 0.812 | 0.817 | 0.19 | 0.622 | 1 |
| **5,000** | **1024** | 0.952 | 0.953 | 0.036 | 0.873 | 1 |
| **10,000** | **128** | 0.313 | 0.292 | 0.171 | 0.017 | 0.656 |
| **10,000** | **256** | 0.671 | 0.749 | 0.201 | 0.15 | 0.923 |
| **10,000** | **512** | 0.879 | 0.879 | 0.062 | 0.789 | 1 |
| **10,000** | **1024** | 0.927 | 0.919 | 0.04 | 0.885 | 1 |
| **50,000** | **128** | 0.252 | 0.23 | 0.175 | 0.006 | 0.686 |
| **50,000** | **256** | 0.714 | 0.768 | 0.175 | 0.152 | 0.964 |
| **50,000** | **512** | 0.889 | 1 | 0.21 | 0.502 | 1 |
| **50,000** | **1024** | 0.96 | 1 | 0.051 | 0.896 | 1 |
| **100,000** | **128** | 0.336 | 0.331 | 0.187 | 0.051 | 0.682 |
| **100,000** | **256** | 0.49 | 0.517 | 0.191 | 0.005 | 0.786 |
| **100,000** | **512** | 0.979 | 1 | 0.026 | 0.947 | 1 |
| **100,000** | **1024** | 0.94 | 0.94 | 0.061 | 0.879 | 1 |
| **full resolution** | **128** | 0.553 | 0.566 | 0.163 | 0.277 | 0.897 |
| **full resolution** | **256** | 0.795 | 0.838 | 0.125 | 0.584 | 0.975 |
| **full resolution** | **512** | 0.851 | 0.851 | 0.093 | 0.635 | 1 |
| **full resolution** | **1024** | 0.939 | 0.928 | 0.048 | 0.81 | 1 |

**Table 11.** Bonferroni-corrected p-values for tests of significant differences between mean correlation coefficients (R^2^) of between PC1 values in replicated alignments of the erinaceomorph dataset.

|  | **5kT**  **128P** | **5kT**  **256P** | **5kT**  **512P** | **5kT**  **1024P** | **10k**  **T128P** | **10kT**  **256P** | **10kT**  **512P** | **10kT**  **1024P** | **50kT**  **128P** | **50kT**  **256P** |
| --- | --- | --- | --- | --- | --- | --- | --- | --- | --- | --- |
| **5kT256P** | 0 |  |  |  |  |  |  |  |  |  |
| **5kT512P** | 0 | 0 |  |  |  |  |  |  |  |  |
| **5kT1024P** | 0 | 0 | 0.018 |  |  |  |  |  |  |  |
| **10kT128P** | 1 | 0.001 | 0 | 0 |  |  |  |  |  |  |
| **10kT256P** | 0 | 0 | 0.015 | 0 | 0 |  |  |  |  |  |
| **10kT512P** | 0 | 0 | 1 | 1 | 0 | 0 |  |  |  |  |
| **10kT1024P** | 0 | 0 | 0.246 | 1 | 0 | 0 | 1 |  |  |  |
| **50kT128P** | 1 | 0 | 0 | 0 | 1 | 0 | 0 | 0 |  |  |
| **50kT256P** | 0 | 0 | 1 | 0 | 0 | 1 | 0.001 | 0 | 0 |  |
| **50kT512P** | 0 | 0 | 1 | 1 | 0 | 0 | 1 | 1 | 0 | 0 |
| **50kT1024P** | 0 | 0 | 0.007 | 1 | 0 | 0 | 1 | 1 | 0 | 0 |
| **100kT128P** | 0.633 | 0.014 | 0 | 0 | 1 | 0 | 0 | 0 | 1 | 0 |
| **100kT256P** | 0 | 1 | 0 | 0 | 0 | 0 | 0 | 0 | 0 | 0 |
| **100kT512P** | 0 | 0 | 0.001 | 1 | 0 | 0 | 0.975 | 1 | 0 | 0 |
| **100kT1024P** | 0 | 0 | 0.068 | 1 | 0 | 0 | 1 | 1 | 0 | 0 |
| **FRT128P** | 0 | 1 | 0 | 0 | 0 | 0.188 | 0 | 0 | 0 | 0.001 |
| **FRT256P** | 0 | 0 | 1 | 0.002 | 0 | 0.092 | 1 | 0.045 | 0 | 1 |
| **FRT512P** | 0 | 0 | 1 | 0.916 | 0 | 0 | 1 | 1 | 0 | 0.025 |
| **FRT1024P** | 0 | 0 | 0.07 | 1 | 0 | 0 | 1 | 1 | 0 | 0 |

**Table 11.** continued

|  | **50kT**  **512P** | **50kT**  **1024P** | **100kT128P** | **100kT256P** | **100kT512P** | **100kT1024P** | **FRT128P** | **FRT**  **256P** | **FRT**  **512P** |
| --- | --- | --- | --- | --- | --- | --- | --- | --- | --- |
| **50kT1024P** | 1 |  |  |  |  |  |  |  |  |
| **100kT128P** | 0 | 0 |  |  |  |  |  |  |  |
| **100kT256P** | 0 | 0 | 0.003 |  |  |  |  |  |  |
| **100kT512P** | 1 | 1 | 0 | 0 |  |  |  |  |  |
| **100kT1024P** | 1 | 1 | 0 | 0 | 1 |  |  |  |  |
| **FRT128P** | 0 | 0 | 0 | 1 | 0 | 0 |  |  |  |
| **FRT256P** | 1 | 0.001 | 0 | 0 | 0 | 0.011 | 0 |  |  |
| **FRT512P** | 1 | 0.442 | 0 | 0 | 0.063 | 1 | 0 | 1 |  |
| **FRT1024P** | 1 | 1 | 0 | 0 | 1 | 1 | 0 | 0.011 | 1 |

**Table 12.** Summary statistics for Mantel tests (p-values) comparing replicated alignments of erinaceomorph dataset.

| **Triangles per Mesh** | **Pseudolandmarks** | **Mean** | **Median** | **Standard Deviation** | **Minimum** | **Maximum** |
| --- | --- | --- | --- | --- | --- | --- |
| **5,000** | **128** | 0.225 | 0.143 | 0.237 | 0 | 0.886 |
| **5,000** | **256** | 0.359 | 0.222 | 0.335 | 0 | 0.933 |
| **5,000** | **512** | 0.419 | 0.435 | 0.41 | 0 | 0.923 |
| **5,000** | **1024** | 0.136 | 0.14 | 0.125 | 0 | 0.521 |
| **10,000** | **128** | 0.36 | 0.297 | 0.3 | 0.001 | 0.956 |
| **10,000** | **256** | 0.336 | 0.197 | 0.316 | 0 | 0.962 |
| **10,000** | **512** | 0.323 | 0.293 | 0.261 | 0 | 0.841 |
| **10,000** | **1024** | 0.14 | 0.052 | 0.176 | 0 | 0.722 |
| **50,000** | **128** | 0.345 | 0.291 | 0.323 | 0 | 0.927 |
| **50,000** | **256** | 0.279 | 0.223 | 0.313 | 0 | 0.999 |
| **50,000** | **512** | 0 | 0 | 0 | 0 | 0 |
| **50,000** | **1024** | 0.008 | 0 | 0.011 | 0 | 0.033 |
| **100,000** | **128** | 0.262 | 0.151 | 0.285 | 0 | 0.94 |
| **100,000** | **256** | 0.367 | 0.278 | 0.364 | 0 | 0.997 |
| **100,000** | **512** | 0.363 | 0 | 0.462 | 0 | 0.959 |
| **100,000** | **1024** | 0.247 | 0.22 | 0.251 | 0 | 0.538 |
| **full resolution** | **128** | 0.332 | 0.11 | 0.382 | 0 | 0.991 |
| **full resolution** | **256** | 0.211 | 0.046 | 0.281 | 0 | 0.93 |
| **full resolution** | **512** | 0.353 | 0.305 | 0.331 | 0 | 0.986 |
| **full resolution** | **1024** | 0.272 | 0.2 | 0.273 | 0 | 0.84 |

**Table 13.** Summary statistics for Robinson-Foulds distances between pairs of phenograms constructed from replicated alignments of the erinaceomorph dataset.

| **Triangles per Mesh** | **Pseudolandmarks** | **Mean** | **Median** | **Standard Deviation** | **Minimum** | **Maximum** |
| --- | --- | --- | --- | --- | --- | --- |
| **5,000** | **128** | 73.5 | 74 | 2.762 | 66 | 78 |
| **5,000** | **256** | 67.722 | 68 | 4.253 | 56 | 74 |
| **5,000** | **512** | 39.222 | 50 | 32.335 | 0 | 70 |
| **5,000** | **1024** | 43.111 | 56 | 25.453 | 0 | 64 |
| **10,000** | **128** | 71.722 | 72 | 3.769 | 64 | 78 |
| **10,000** | **256** | 70 | 70 | 2.986 | 64 | 74 |
| **10,000** | **512** | 52 | 56 | 17.521 | 0 | 68 |
| **10,000** | **1024** | 48.167 | 57 | 22.113 | 0 | 62 |
| **50,000** | **128** | 66.111 | 66 | 4.139 | 54 | 74 |
| **50,000** | **256** | 61.278 | 63 | 4.903 | 44 | 68 |
| **50,000** | **512** | 13.333 | 0 | 25.298 | 0 | 60 |
| **50,000** | **1024** | 17.111 | 0 | 21.754 | 0 | 44 |
| **100,000** | **128** | 68.722 | 68 | 4.145 | 60 | 78 |
| **100,000** | **256** | 64.778 | 64 | 5.622 | 50 | 74 |
| **100,000** | **512** | 22.556 | 0 | 28.704 | 0 | 60 |
| **100,000** | **1024** | 26 | 26 | 26.369 | 0 | 52 |
| **full resolution** | **128** | 57.611 | 60 | 6.587 | 38 | 66 |
| **full resolution** | **256** | 55.611 | 55 | 5.233 | 44 | 64 |
| **full resolution** | **512** | 49.222 | 52 | 11.051 | 0 | 64 |
| **full resolution** | **1024** | 34.833 | 42 | 17.464 | 0 | 56 |

**Table 14.** Summary statistics for correlation coefficients (R^2^) between PC1 values for pairs of replicated alignments of the *Mus musculus* dataset.

| **Pseudolandmarks** | **Mean** | **Median** | **Standard Deviation** | **Minimum** | **Maximum** |
| --- | --- | --- | --- | --- | --- |
| **399** | 0.815 | 0.818 | 0.118 | 0.531 | 1 |
| **600** | 0.476 | 0.358 | 0.379 | 0.008 | 1 |
| **1000** | 0.555 | 0.599 | 0.343 | 0.025 | 0.997 |
| **2000** | 0.579 | 0.621 | 0.304 | 0.036 | 1 |
| **3000** | 0.911 | 0.903 | 0.056 | 0.801 | 0.998 |

**Table 15.** Summary statistics for Mantel tests (p-values) comparing replicated alignments of *Mus musculus* dataset.

| **Pseudolandmarks** | **Mean** | **Median** | **Standard Deviation** | **Minimum** | **Maximum** |
| --- | --- | --- | --- | --- | --- |
| **399** | 0 | 0 | 0 | 0 | 0 |
| **600** | 0 | 0 | 0 | 0 | 0 |
| **1000** | 0 | 0 | 0 | 0 | 0 |
| **2000** | 0 | 0 | 0 | 0 | 0 |
| **3000** | 0.085 | 0.009 | 0.123 | 0 | 0.499 |

**Table 16.** Summary statistics for Robinson-Foulds distances between pairs of phenograms constructed from replicated alignments of the *Mus musculus* dataset.

| **Pseudolandmarks** | **Mean** | **Median** | **Standard Deviation** | **Minimum** | **Maximum** |
| --- | --- | --- | --- | --- | --- |
| **399** | 19 | 21 | 7.368 | 0 | 28 |
| **600** | 18.222 | 19 | 5.488 | 2 | 26 |
| **1000** | 19.333 | 20 | 5.596 | 8 | 30 |
| **2000** | 19.167 | 20 | 6.492 | 0 | 28 |
| **3000** | 11.056 | 12 | 4.647 | 2 | 18 |
